# Ecological and Evolutionary Effects of Stickleback on Community Structure

**DOI:** 10.1371/journal.pone.0059644

**Published:** 2013-04-03

**Authors:** Simone Des Roches, Jonathan B. Shurin, Dolph Schluter, Luke J. Harmon

**Affiliations:** 1 Department of Biological Sciences, University of Idaho, Moscow, Idaho, United States of America; 2 Division of Biological Sciences, University of California San Diego, San Diego, California, United States of America; 3 Biodiversity Research Centre, University of British Columbia, Vancouver, British Columbia, Canada; University of San Diego, United States of America

## Abstract

Species’ ecology and evolution can have strong effects on communities. Both may change concurrently when species colonize a new ecosystem. We know little, however, about the combined effects of ecological and evolutionary change on community structure. We simultaneously examined the effects of top-predator ecology and evolution on freshwater community parameters using recently evolved generalist and specialist ecotypes of three-spine stickleback (*Gasterosteus aculeatus*). We used a mesocosm experiment to directly examine the effects of ecological (fish presence and density) and evolutionary (phenotypic diversity and specialization) factors on community structure at lower trophic levels. We evaluated zooplankton biomass and composition, periphyton and phytoplankton chlorophyll-*a* concentration, and net primary production among treatments containing different densities and diversities of stickleback. Our results showed that both ecological and evolutionary differences in the top-predator affect different aspects of community structure and composition. Community structure, specifically the abundance of organisms at each trophic level, was affected by stickleback presence and density, whereas composition of zooplankton was influenced by stickleback diversity and specialization. Primary productivity, in terms of chlorophyll-*a* concentration and net primary production was affected by ecological but not evolutionary factors. Our results stress the importance of concurrently evaluating both changes in density and phenotypic diversity on the structure and composition of communities.

## Introduction

Evidence that species’ phenotypic diversity and composition influence the structure of ecosystems is accumulating [1], [2], [3], [4], [5]. Until recently, however, this work has mostly ignored contemporary evolution, presuming that ecological and evolutionary processes occur over dramatically different time scales [6], [7]. Increasing evidence for rapid evolution over the order of a few generations [6], [8], [9], [10], [11], [12] has made it clear that ecological and evolutionary time scales overlap broadly [6], [9], [13], [14], [15], [16], and that both ecological and evolutionary factors can have strong effects on communities, even over short periods of time. For example, ecological studies show how predator presence and density influence lower trophic levels [17], [18], [19], [20], [21]. Evolutionary studies demonstrate how trophic ecology within a predator population is affected by among-population variation in life history [22], [23], age structure [23], and ontogeny [24]. A few studies have further characterized the dynamic feedback loops between evolutionary diversification and ecosystem properties, such as community structure and organization [3], [9], [25], [26].

Although much current work focuses on whether or not interactions between ecology and evolution occur, some studies have begun to explore the quantitative effects of eco-evolutionary dynamics [15] using mathematical models (e.g. [27], [28]). Such models require detailed quantitative information about the relative magnitude of both ecological and evolutionary changes on the structure of communities. Here we examine the community-wide effects of a top predator, the three-spine stickleback (*Gasterosteus aculeatus*), on organisms at lower trophic levels that result from its presence and density (ecology) and its specialization and speciation (evolution). Recent work by Harmon et al. [3] has shown the importance of stickleback speciation and trophic specialization on ecosystem parameters. Here we add to the findings of that research by evaluating the importance of these evolutionary effects in the context of the potentially larger effects of changing stickleback density. Specifically, we simultaneously investigate ecological (fish presence and density) and evolutionary (phenotypic diversity and specialization) effects on community structure and composition. The goal of our study was to compare the magnitude of change in community structure driven by evolutionary diversification (recently shown by Harmon et al. [3]) to that brought about by differences in fish density due to the well-established mechanisms of trophic cascades.

The threespine stickleback is a model organism for evolutionary and ecological research (e.g. [29], [30], [31], [32], [33], [34]). Marine stickleback probably invaded coastal lakes in British Columbia, Canada, between 10–12,000 years ago at the end of the last ice age [34]. Most colonists gave rise to solitary populations of generalist ecotypes that opportunistically feed in both limnetic (open-water) and benthic (lake-bottom) habitats. In a few “species pair” lakes two ecologically divergent, reproductively isolated ecotypes exist in sympatry [32], [34]: a limnetic type with a narrow gape, many long gill rakers and a slender body, and a benthic type with a large gape, few short gill rakers, and a deep body [34]. The morphological features that differ between the two types improve feeding performance in their respective niches [35]. Limnetic stickleback feed primarily on zooplankton in the open-water, whereas benthic stickleback consume larger invertebrates from the lake-bottom [36]. Individuals of each type grow most rapidly in their respective habitats [35]. Diversification in the species pair lakes probably arose from double colonization followed by character displacement, whereby the first colonist evolved into the benthic ecotype, and the second became confined to the limnetic niche [34].

Researchers have shown that stickleback can affect community structure via both their ecology [17] and evolution [3]. For example, the limnetic stickleback, like other zooplanktivorous predators (see [18], [37], [38]), affect the pelagic food chain through cascading trophic interactions [17] in which they increase primary productivity by reducing the abundance of herbivorous zooplankton. Other studies using mesocosms have shown evolutionary diversification and specialization of stickleback traits affect both biotic (invertebrate abundance, and phytoplankton chlorophyll-*a* concentration) and abiotic components (attenuation of light through the water column) of the surrounding ecosystem [3]. Like stickleback, the density and diversity of guppies [1] and alewives [25], [26] also influences their ecological surroundings. In all cases, the effects of fish density and diversity are a result of classical trophic interactions [1], [25], [26] and/or the liberation of nutrients by excretion [1].

In this paper, we examine whether zooplankton abundance and composition, benthic and limnetic chlorophyll-*a* concentration and dissolved oxygen were affected by changes in stickleback ecology and evolution concurrently. We use these measures to represent the biotic components of community structure that are most commonly mediated by a top-predator via trophic cascades or nutrient liberation. Previous research has shown that changes in density [17] and evolutionary diversification and specialization of stickleback can affect these aspects of community structure [3]. Here we describe a study carried out before a previously published experiment by Harmon *et al.* [3] in which we simultaneously quantified and compared the ecological (fish presence and density) and evolutionary (phenotypic diversity and specialization) effects of stickleback on community structure using a mesocosm experiment.

We make the following predictions for the ecological and evolutionary effects of stickleback based on the findings of previous research [1], [3], [17]. In general, for ecological effects, increasing predation brought about by higher fish density will decrease large zooplankton biomass. Smaller inedible microzooplankton, such as copepod nauplii and rotifers, will be liberated from competition with the larger species and therefore increase in biomass. Finally, primary producers (phytoplankton and periphyton) and thus primary productivity (dissolved oxygen concentration) will increase due to reduced herbivory by large zooplankton species and through nutrient liberation by foraging stickleback. For evolutionary effects, we expect the magnitude of differences between treatments will be less pronounced due to opportunistic feeding behaviors of all fish and strong linkages between the “limnetic” and “benthic” habitats. In general, limnetic fish should have the strongest affect on large zooplankton, followed by generalist, and then benthic fish. As described above, smaller microzooplankton species should increase in the absence of larger species. Finally, because we expect large zooplankton species to graze both in the limnetic and benthic habitats, we predict both periphyton and phytoplankton (and dissolved oxygen concentration) to increase in the presence of limnetic fish.

## Materials and Methods

### Mesocosm Construction

We collected stickleback from wild freshwater populations on Texada Island, British Columbia, Canada (British Columbia Ministry of the Environment Collection Permit No.: NA/SU06-21454), thus all phenotypic variation we attribute to “evolutionary” differences is also that which is present in natural systems. We caught specialist limnetic and benthic individuals from Paxton Lake, and generalist individuals from a solitary population in Cranby Lake. We collected and used only female fish in the experiment because within the ecotypes, females exhibit the most specialized feeding behaviors; male limnetics, for example, will opportunistically feed in the littoral zone during the spring when they are nesting [35]. We used disinfected metal minnow traps and dip nets and transported the fish to the University of British Columbia campus, Vancouver, Canada, where we housed them in 20 gallon glass aquariums. We euthanized fish using MS-222 throughout the experiment only when they showed signs of physiological stress. We housed fish that survived the experiment in laboratory aquariums indefinitely. Our study was carried out in accordance with the Canadian Council of Animal Care Guidelines and approved by the University of British Columbia Animal Care Committee (Protocol Number: A04-0208).

We performed the experiment from 25 May to 17 July 2006 at the University of British Columbia in cattle tank mesocosms. Although community responses in mesocosms only provide a window into what occurs in natural systems and the inferences can be made from their use in ecological experiments are limited [39], they allow us to compare treatments with high levels of replication and control. Our mesocosms had a maximum volume of 1136 L (approximate dimensions L: 1.61 m, W: 1.75 m, H: 0.64 m). Prior to the addition of water we added approximately 30 liters of loosely packed of leaf litter and benthic sediments per tank. We collected the litter and sediments from a nearby fish-less experimental pond to provide an inoculum for the initial community of invertebrates, plankton, macrophytes, dead organic matter and detritus. We used inoculum from a fish-less pond to simulate an ecosystem prior fish colonization. We filled the tanks with well water to approximately 20 cm below the rim and let the water sit for one week before adding fish. Before we added fish, we fertilized the tanks with NaNO_3_ (2.46 g per tank) and NaH_2_PO_4_ (0.18 g per tank; [40]) to boost initial primary productivity.

### Fish Treatments and Surveys

To test the effects of top predator ecology and evolution on aquatic community structure, we manipulated both fish density and phenotypic diversity across six mesocosm treatments. We divided forty tanks into the six treatments as follows: no fish control (NF, six tanks); generalist fish only (G, eight tanks); limnetic fish only (L, six tanks); benthic fish only (B, six tanks); both benthic and limnetic fish (BL, eight tanks); and benthic and limnetic fish at twice the fish biomass (g of fish per L) as the other fish treatments (BBLL, six tanks). We used more replicates of the G and BL treatments to make use of all available tanks and all our statistical analyses allowed for this unequal replication. All single density tanks (G, B, L, BL) had a summed fish weight between 3.0 and 3.5 g, while the double density tanks (BBLL) had a total fish weight of 6.0 to 7.0 g. Tanks from different treatments contained different total numbers of fish (L treatment: four fish, B treatment: two fish, G treatment: three fish, BL treatment: three fish, one benthic and two limnetics, BBLL treatment: six fish, two benthics and four limnetics). Adult benthic individuals are roughly double the weight of limnetics, whereas generalist individuals are intermediate between the two. Furthermore, natural populations of stickleback in Paxton Lake contain approximately two limnetic individuals for every benthic fish [41]. In all treatments, we used fish densities of two to four fish per 2.40 square meters in diameter, which are within the ranges found in natural populations [19], [42], [43] including those found in Paxton Lake, which contains densities from fewer than one [41] to up to 28 [43] fish per square meter of water surface area. Our experimental densities were also comparable to those used in recent studies of intraspecific competition in stickleback [44], [45]. Finally, evidence suggests that there is a strong link between body size and prey consumption in other species of stickleback [46]; this trend is also apparent, however is less pronounced, for the threespine stickleback species pairs [47].

We arranged the tanks in four rows of 10. We divided the 40 tanks into six spatially clustered blocks, four of which contained six tanks and two of which contained eight. We arranged the blocks from bottom to top across the columns. We randomly assigned all six treatments to the tanks within each block (NF, G, B, L, BL, BBLL), with the two blocks of eight tanks assigned one additional replicate of both the G and BL treatments.

We weighed and measured all 120 fish before adding them to the tanks on 25 May 2006. Because all fish treatments experienced some mortality throughout the experiment, we systematically surveyed for living and dead fish three times weekly. We replaced a total of 40 dead fish (33% of the total number) as soon as possible with fish of similar mass to maintain the top predator biomass at a constant level, while minimizing the potential affects of fish decay on ecosystem variables. We recovered all fish at the termination of the experiment using minnow traps, anesthetized them with MS222 and preserved them in 95% ethanol.

### Sampling

We sampled invertebrates (including both edible large zooplankton species, and smaller, inedible microzooplankton species) from the water column one month after the addition of fish to the mesocosms. We took water samples using a 10 cm diameter PVC pipe that could be sealed at the bottom with a tennis ball attached to a string. This apparatus allowed us to sample planktonic organisms throughout the water column at a volume equal to ∼1 L. We took samples from both the periphery and center of the tank and emptied them into a bucket until we obtained a total volume of about 11 L. We filtered the sample water through a 54 µm sieve to concentrate zooplankton and other planktonic invertebrates, which we then stained and fixed with Lugol’s Iodine solution. We identified zooplankton under a stereo-microscope at 3.2 times magnification. We attempted to identify individuals to genus; however, in some cases (e.g. cyclopoid copepods), we identified individuals to sub-order. We estimated the average biomass of each taxon by taking the mean lengths of 30 haphazardly selected individuals and applying length-weight regression [48]. To estimate total community biomass per liter, we multiplied average genus weights by total invertebrate population densities.

We measured standing stock of both the limnetic (phytoplankton) and benthic (periphyton) primary producers by chlorophyll-*a* concentration. We collected 100 mL water samples from each tank within three days of invertebrate sampling for estimating phytoplankton chlorophyll-*a* concentration. On the same day we suction filtered these samples through GF/C 24 mm Whatman glass microfiber filters. We then cold-extracted each filter in 10 mL of 90% acetone in darkness overnight before measuring fluorescence using a Trilogy Fluorometer (model 7200-000). We sampled periphyton growth on unglazed ceramic tiles (26 cm^2^ area; [49]), which we had added to all tanks prior to the addition of fish. We removed a tile from the bottom of each tank one month after fish were added and scraped the algae from the entire surface of the tile with a nylon brush into 100 mL of distilled water. We filtered and measured chlorophyll-*a* from the periphyton samples in acetone using the same method as for phytoplankton.

We also measured net primary productivity (NPP) in the mesocosms by recording daily dissolved oxygen cycles with an YSI DO2 Probe (model 55) at sunset and sunrise of the same night. We measured instantaneous DO_2_ for all 40 tanks over a 30 to 45 minute period surrounding sunset and sunrise. We estimated NPP for the system by taking the difference in dissolved oxygen concentration between sunset and sunrise for a given date. Thus we can estimate the quantity of oxygen produced by all photosynthetic organisms for the entire tank minus the respiration of all organisms [50].

### Data Analysis

We compared differences in zooplankton biomass and composition, and primary productivity among the six different treatments before performing planned contrasts for our specific hypotheses regarding stickleback ecology (density) and evolution (diversity, specialization). We used one-way ANOVAs to evaluate differences in zooplankton dry biomass (mg/L) for both large, edible zooplankton (such as cladocerans and copepods) and inedible microzooplankton (rotifers and copepod nauplii) among treatments. To examine the effect of different treatments on zooplankton community structure, we performed MANOVA (multivariate analysis of variance) on two-dimentional nonmetric multidimentional scaling (NMDS) using each zooplankton genus’ total normalized dry biomass per tank (mg/L). For NMDS we used Bray-Curtis distances and retained two axes [51], which represent the species with the strongest positive and negative loadings. We plotted the species loadings of these axes to determine the species that explained the most variation among treatments (see Figure S1). We performed the same planned contrasts described above to determine differences in zooplankton community composition between pairs of treatments. For productivity measures, we used one-way ANOVAs to determine differences periphyton chlorophyll-*a* concentration (µg/cm^2^), phytoplankton chlorophyll-*a* concentration (in µg/L), and dissolved oxygen concentration (mg/L). All our ANOVAs included possible interactions of treatments with block effects.

We used Welch’s t-tests to test our specific hypotheses regarding the effects of changing stickleback density and diversity on community structure. We performed two planned contrasts to evaluate the effects of stickleback ecology: the effects of lake colonization by a generalist (NF/G), and the impact of increasing density of specialists (BL/BBLL). We performed the planned contrasts G/BL, G/L, and G/B (adapted from [3], [52]) to evaluate effects of stickleback evolution. These contrasts focus on the community-wide effects of evolutionary changes in stickleback: the first tests the effects of diversification from a generalist to two specialists, the second and third test the effects of trophic specialization. We did not correct for multiple comparisons since all contrasts were planned; instead, we set alpha = 0.05 for all comparisons. We calculated effect sizes to determine the degree of response of our measured ecological parameters between planned treatment comparisons [53]. For any given contrast, we measured effect size as ln[(mean treatment_1)/(mean treatment_2)] [54]. Therefore, large effect sizes for NF/G or BL/BBLL indicated a strong effect of increasing fish density or presence and large effect sizes for contrasts between G/BL, G/B and G/L indicated a strong effect of diversity or specialization.

## Results

Differences in stickleback ecology, evolution or both affected nearly all parameters measured. Whereas differences in stickleback density (ecology effects) had larger effects on the abundance of organisms at lower trophic levels, stickleback diversity and specialization (evolutionary effects) mainly affected zooplankton community composition.

**Figure 1:**
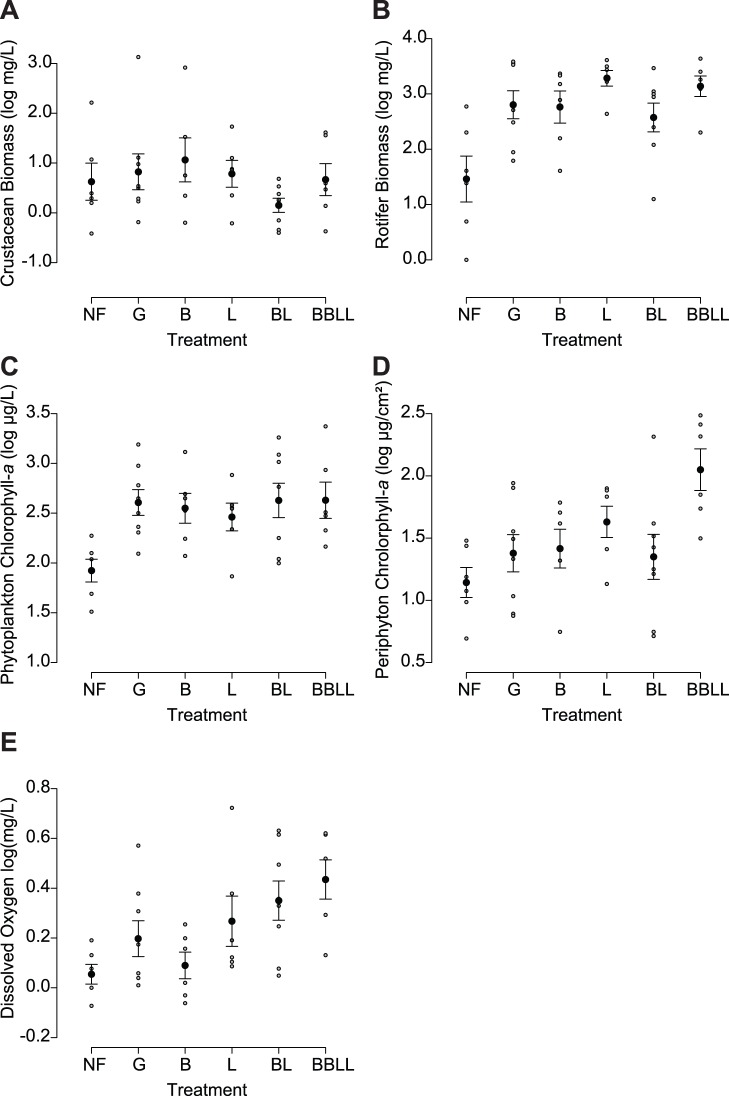
Zooplankton mass in grams per liter across different treatments for (A) crustaceans and (B) rotifers; primary producer abundance in terms of (C) concentration of periphyton and (D) phytoplankton chlorophyll-*a* concentration in milligrams per liter across treatments and total system net productivity (E) in terms of daily changes in dissolved oxygen concentration in milligrams per liter across treatments. Bars represent standard error of the mean. Treatments correspond to NF = no fish, G = generalist ecotype, B = benthic ecotype, L = limnetic ecotype, BL = limnetic and benthic ecotype together, BBLL = double density of limnetic and benthic ecotype together.

Zooplankton response to changes in stickleback ecology and evolution was not as strong as predicted. Total large zooplankton biomass did not differ among treatments (ANOVA, treatment effect: F_5, 28_ = 2.5; P>0.05, block effect: F_1, 28_ = 5.7; P<0.05, no interaction effect, Figure 1A), thus demonstrating no significant effects of either stickleback ecology or evolution. Here, the largest effect size was between the generalist (G) and benthic-limnetic (BL) treatments. Microzooplankton biomass, however, differed significantly among treatments (ANOVA, treatment effect: F_5, 28_ = 2.4; P = 0.05, block effect: F_5, 28_ = 2.2; P>0.05, no interaction effect, Figure 1B); however, only one planned contrast, which tested the effects of stickleback ecology, was marginally significant (all others P>0.05): the generalist (G) treatment had higher biomass of microzooplankton than the no fish (NF) treatment (planned contrast t-test: t_10.3_ = −2.13, P = 0.02, Table 1). Furthermore, this comparison had the largest effect size of all planned contrasts. Overall, microzooplankton effect sizes for different stickleback ecology comparisons (NF/G and BL/BBLL) were higher than those for different stickleback evolution comparisons (G/BL, G/B and G/L; Table 1).

**Table 1:** Test statistics and effect sizes for planned contrasts showing the importance of stickleback ecology and evolution on different community parameters.

Ecosystem Parameter	Test Statistic	Planned Contrast Effect Sizes				
		Ecological		Evolutionary		
		G/NF	BL/BBLL	G/BL	G/B	G/L
Crustacean biomasslog(mg/L)	F_5, 28_ = 1.0	0.46	−0.70	1.27	−1.33	0.53
Rotifer biomasslog(mg/L)	F_5, 28_ = 2.8*	1.16†	−0.45	0.24	0.07	−0.32
Periphyton chlorophyll concentrationlog(µg/L)	F_5, 28_ = 2.8*	0.71††	0.01	−0.06	0.06	0.16
Phytoplankton chlorophyll concentrationlog(µg/L)	F_5, 28_ = 3.8††	0.28	−0.64†	−0.16	−0.01	−0.21
Dissolved oxygen concentrationlog(mg/L)	F_5, 28_ = 3.6††	0.16	−0.08	−0.15	0.12	−0.08

Larger effect sizes correspond to responses of larger magnitude.

* P<0.1,

† P<0.05,

†† P<0.01.

**Figure 2:**
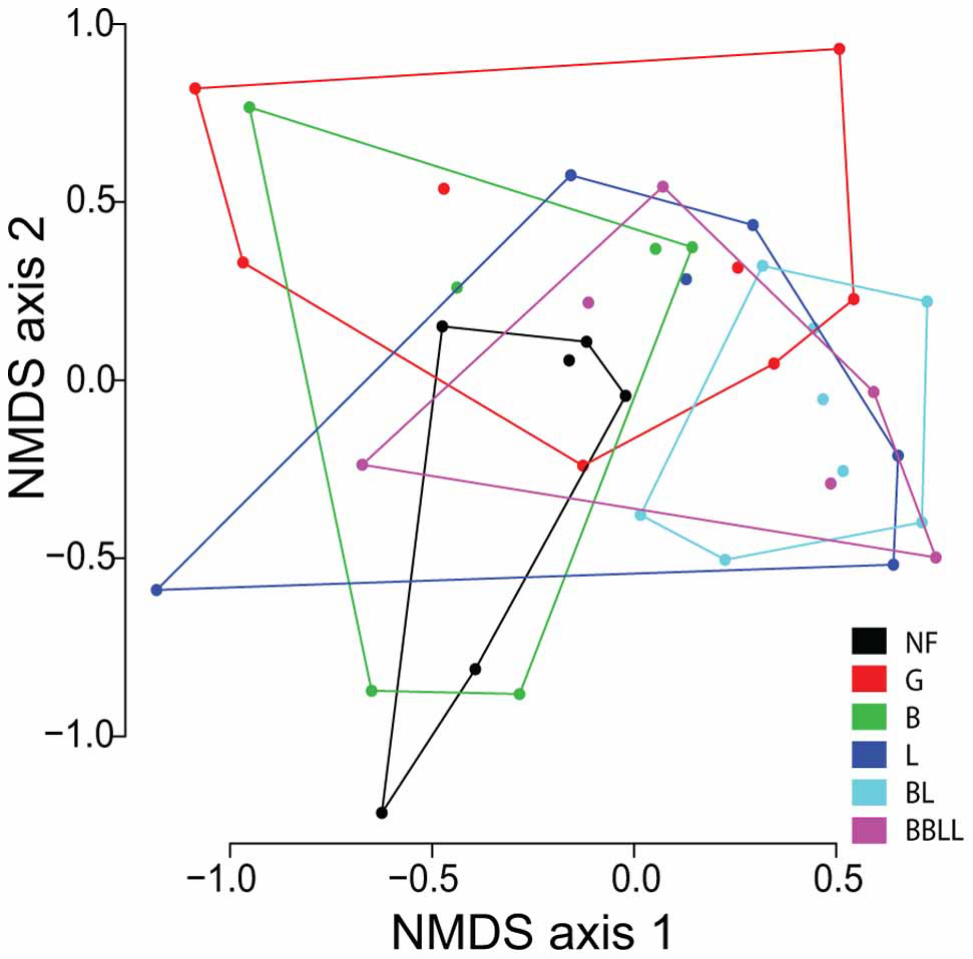
The first two non-metric multidimensional scaling (NMDS) axes for zooplankton community composition. Points represent individual tanks, colors represent treatments (NF = no fish, G = generalist ecotype, B = benthic ecotype, L = limnetic ecotype, BL = benthic and limnetic ecotype together, BBLL = double density of benthic and limnetic ecotype together), and polygons surround all tanks of a given treatment. The numbers on each axis correspond to the genera of zooplankton with the strongest loadings (negative and positive). For a graphical representation of the zooplankton genera loadings, please refer to Figure S1.

Community composition of zooplankton (both larger zooplankton and microzooplankton) was influenced by stickleback treatment (MANOVA, Wilk’s _l5, 28_ = 0.51, P = 0.03, Figure 2). Our NMDS demonstrated strong positive loadings for *Diaphanosoma* and negative loadings for *Daphnia* on axis 1, and strong positive loadings for *Daphnia* and negative loadings for *Chydoras* on axis 2. Planned contrasts indicated significant differences in composition for the comparison between the generalist (G) and benthic-limnetic (BL) treatments (planned contrast t-test, t_30_ = 11.6, P = 0.02), demonstrating an effect of stickleback evolution. These treatments differed most drastically on NMDS axis 2, with the BL treatment tanks showing less variation, with a higher proportion of diaphanosoma, and a smaller proportion of daphnia than the G treatments. All other comparisons were insignificant (P>0.05).

Differences in stickleback ecology affected both benthic and limnetic primary producers to a greater extent than differences in stickleback evolution. Periphyton chlorophyll-*a* concentration was significantly different among treatments (ANOVA, F_5, 28_ = 2.8, P = 0.03, Figure 1C). The generalist (G) treatment had significantly higher periphyton chlorophyll-*a* concentration when compared the no fish (NF) treatment (planned contrast t-test: t_12.0_ = −2.76, NF/G, P = 0.002, Table 1), demonstrating an effect of differences in stickleback density. This comparison also had the largest effect size. All other planned comparisons, however, were nonsignificant (P>0.05). Phytoplankton chlorophyll-*a* concentration also differed significantly among treatments (ANOVA, F_5, 28_ = 3.8, P = 0.008, Figure 1D), and only the planned contrast between the single and double density benthic-limnetic treatments was significant (planned contrast t-test, t_11.9_ = −2.84, BL/BBLL, P = 0.02, Table 1), showing an effect of stickleback density. Phytoplankton chlorophyll-*a* effect sizes were generally higher for ecological comparisons (Table 1), with the BL/BBLL effect size being the largest. Finally, NPP was significantly different among treatments (ANOVA, F_5, 34_ = 3.6; P = 0.01, Figure 1E). However, our planned contrasts did not include comparisons that were significant. The effect sizes for NPP were comparable among treatments differing in stickleback ecology and evolution (Table 1).

Our treatment tanks experienced consistent fish mortality throughout the experiment. Although the mean recovery for the different treatments was only 62% of the fish, we found approximately equivalent proportions of total missing fish from each treatment at the end of the experiment (Chi Squared Test, X^2^_7_ = 21, P = 0.2, Table S1). Furthermore, we replaced equivalent proportions of each ecotype throughout the experiment (Chi Squared Test X^2^_7_ = 21, P = 0.2, Table S1). These results suggest that fish mortality did not differ across treatments throughout the study and at the termination of the experiment, so that fish mortality does not explain differences among treatments in our experiment (also see [3]).

## Discussion

Ecological and evolutionary changes in top-predator density and diversification, respectively, may occur simultaneously upon colonization of a new environment. Although research has shown the importance of both these factors, less is known about the magnitude of community-wide change brought about by one or the other. In our experiment we showed that both ecological differences in density and evolutionary differences in diversity of lake stickleback can have independent and immediate effects on the surrounding community. In particular, our results show that fish density affects the relative abundance of organisms at lower trophic levels to a higher degree than fish diversity. On the other hand, fish diversity more strongly influences community structure of prey items.

Nearly all community parameters measured (zooplankton biomass and composition, periphyton and phytoplankton chlorophyll-*a* concentration, net primary productivity) were affected by either density or diversity of stickleback. Although we acknowledge that the inferences we make from our analyses are limited based on our decision to use an alpha value of 0.05 with multiple planned contrasts, our results are consistent with numerous other studies that describe how predators affect community structure of organisms at lower trophic levels (e.g. [17], [18], [19], [20], [21], [22], [23]). Specifically, our study relates closely to the work of Harmon *et al.* [3], which provided evidence for multiple community-level effects that we also documented here. Our results expand on these findings by showing that that fish density has a greater impact than fish diversity on these aspects of community structure.

Changes in density of stickleback largely influenced the abundance of organisms at lower trophic levels. The most notable exception was the biomass of large zooplankton species, which did not differ significantly among treatments. Previous research has shown that the initial effect of stickleback is a shift in zooplankton community structure towards smaller bodied, inedible microzooplankton species [17] such as copepod nauplii and rotifers. In our study, however, large bodied, edible zooplankton (such as *Daphnia*, and calanoid copepods), were generally in low abundance across all treatments. The absence of these larger species, which are usually selectively predated on by fish, may explain why we observed no differences between density treatments. Despite the lack of response in large zooplankton biomass, we did record a small increase in biomass of microzooplankton (such as *Polyarthra* and *Keratella*) in generalist stickleback treatments compared to no fish treatments (Figure 1A–B, Table 1), a contrast which also had the largest effect size. We have no evidence of stickleback selectively foraging on large zooplankton and liberating microzooplankton from competition [34], [35] as the former do not show significant differences among treatments. Therefore, a more likely explanation for an increase in microzooplankton biomass is the regeneration of nutrients through fish excretion [55], [56] or mortality [56] stimulating primary production and thus microzooplankton grazing. However, because overall mortality did not differ among treatments, we do not attribute microzooplankton biomass to differential fish death; instead, we suggest variation in abundance among treatments is related to more complex changes in nutrient cycling caused by differences in fish traits across treatments, as described below.

Stickleback diversity caused a significant change in zooplankton community composition (Figure 2), similar to results found by Post et al. [26]. Treatments varied most in their presence of *Diaphanosoma* (prominent in benthic-limnetic treatments) and *Daphnia* (prominent in generalist treatments). Indeed, a significant difference in zooplankton community composition was only found between the generalist and benthic-limnetic treatments. Of these, the generalist treatments represented a wide variety of zooplankton communities, whereas the benthic-limnetic treatments commonly had high abundance of *Diaphanosoma* and lower abundance of *Daphnia*. The observed shift may have been driven by opportunistic feeding behaviors of the generalist type, causing the reduction in the most abundant zooplankton species, whereas specialist feeding behaviors may have been dictated by competition-mediated character displacement [36]. Future studies that sample multiple times throughout the experiment could examine the possibility of this mechanism. Finally, although zooplankton biomass did not differ significantly between the generalist and benthic-limnetic treatments, changes in zooplankton body size may have been an important response to specialized predation by benthic and limnetic fish.

Stickleback density had varying effects on periphyton and phytoplankton chlorophyll-*a* concentration (Figure 1C–D). In both cases, there were significant differences among treatments with ecological contrasts having higher effect sizes than evolutionary contrasts. Periphyton chlorophyll-*a* concentration was higher in the presence of generalist stickleback than in the no fish treatment (Figure 1C, Table 1), but non-significant for all other contrasts. Increased chlorophyll-*a* concentration in the benthic environment may have resulted from strong linkage between limnetic and benthic communities in the small mesocosm environment. By consuming invertebrate grazers, fish liberate the nutrients from sediments and invertebrate biomass to a dissolved form, useable by periphyton [57], [58]. The lack of differences in periphyton growth among all fish treatments could be a result of generalist and limnetic fish opportunistically feeding in the benthos and coupling the two habitats [59]. Phytoplankton chlorophyll-*a* concentration was not significantly different between no fish and the generalist fish treatment (Figure 1D, Table 1), contrary to results in other experiments [17], [37], [38], [60]. However, phytoplankton chlorophyll-*a* concentration was significantly higher in the double density treatment when compared to the single density benthic/limnetic treatment (Table 1). A higher density of fish may be required for observable differences in limnetic productivity (see [3], where a higher stickleback biomass used per volume led to differences in limnetic productivity). Competitor-driven feeding behaviors of stickleback [36] may have caused limnetic fish to become more specialized in their resource consumption and cause a stronger trophic cascade in the limnetic food chain. Finally, differences in stickleback numbers used in the treatments (four limnetics/two benthics/three generalists) may have influenced the liberation of nutrients via excretion; however, because periphyton and phytoplankton chlorophyll-*a* concentration did not differ among these treatments, it is unlikely this influenced our results. Harmon *et al.* [3] present more detailed evidence for the mechanism behind how dissolved nutrient levels may have influenced primary productivity in our mesocosm experiments.

Stickleback density and diversity also affected net system primary productivity as measured by daily oxygen cycles (Figure 1E, Table 1). Although none of our planned contrasts showed significant differences in net primary production, dissolved oxygen appeared to increase gradually from our no fish treatment, to our single density treatments, to our double density treatment (Figure 1E). Furthermore, all treatments with limnetic fish appeared to have higher primary productivity levels than the no fish control, although we did not evaluate this comparison directly (Figure 1E). It may be that specialization or introduction of a limnetic top predator could have a large effect on entire system primary productivity. Other evidence supports the importance of diversity for entire system primary productivity, and demonstrates a marginally significant difference between benthic-only and benthic/limnetic treatment primary productivity [3]. Harmon *et al.* [3] suggest dissolved organic carbon composition and attenuation of light can be strongly altered by the diversity and specialization of stickleback. Because we did not measure these same physical attributes in our mesocosms, we cannot evaluate how different densities and diversities of stickleback may influence these abiotic attributes of the ecosystem; however, it is likely that stickleback density has a large role in ecosystem function as has been shown for other fish (e.g. [1], [61], [62]). Changes in food chain length can shift the carbon balance between water bodies and the atmosphere from positive to negative [59]. Our results indicate that changes in the mean value and variance of phenotypic traits among predators can also have substantial impacts on the rate of carbon loss or uptake by freshwater ponds.

We observed smaller effects of stickleback evolution on community structure than Harmon *et al.* [3]. We attribute these differences to a lower density of stickleback used in the current study, which was performed prior to the Harmon *et al.* experiment. For example, our single density treatments contained a total fish biomass of between 3.0 and 3.5 g, where as Harmon *et al.* [3] had a constant density of between 5.0 and 6.0 g (see supplementary material). As such, competition was likely very severe only in our double density treatment where resources were more limited [45]. Density is often more important than diversity across predator clades in influencing ecosystem function [63]; indeed, only at high predator densities does resource partitioning make phenotypic diversity important [45]. The importance of these observations should be supported by further investigation into whether species pair lakes contain a higher density of stickleback than solitary species lakes, and if so, whether this is a result of resource partitioning. Furthermore, although several studies have shown that stickleback have ecological effects in ponds and mesocosms, it remains to be shown whether these extend to natural lake environments. Future studies comparing the effects of stickleback diversity and density on community composition and ecosystem processes in lakes would provide insight into how extensively we can apply our results to natural systems.

When evolution of species is rapid, its effects on the surrounding environment can be closely tied to ecology [15], [16]. Although ecosystem function is frequently related to the overall effect of biodiversity across lineages [63], [64], [65], the level of diversity within a lineage is less commonly shown to influence ecological dynamics. Diversification within a lineage in novel environments can occur rapidly, such as over a few generations, and be accompanied by changes in density and phenotypic diversity [10], [66], [67], [68]. Our results suggest that ecological factors (stickleback presence and density) have a more prominent impact on community abundance, whereas evolutionary factors (speciation and specialization) more strongly influences community composition. Although predator presence and density may cause more obvious changes in abundance of organisms at lower trophic levels via trophic cascades, phenotypic diversity may have more subtle effects on community composition as a result of trophic specialization.

## Supporting Information

Figure S1 **Loadings plotted on NMDS axes 1 and 2 demonstrating the zooplankton genera responsible for the most variation in community composition across treatments.**
(EPS)

Table S1 **Percent replacement (of deceased/sick fish with new fish throughout experiment) and recovery (total number of fish collected at end of experiment) of each fish ecotype in each treatment.**
(DOCX)
