# Supplementary material for: Ecological and Evolutionary Effects of Stickleback on Community Structure
Source: PLoS One. 2013 Apr 3;8(4):e59644. doi: 10.1371/journal.pone.0059644 (PMC3616105; doi:10.1371/journal.pone.0059644)
Supplement: Table S1 — Percent replacement (of deceased/sick fish with new fish throughout experiment) and recovery (total number of fish collected at end of experiment) of each fish ecotype in each treatment. (DOCX) [file pone.0059644.s002.docx]

**Electronic Supplementary Material**

Table S1: Percent replacement (of deceased/sick fish with new fish throughout experiment) and recovery (total number of fish collected at end of experiment) of each fish ecotype in each treatment.

| Treatment | Ecotype | Percent Replaced | Percent Recovered |
| --- | --- | --- | --- |
| G | G | 25 | 63 |
| B | B | 25 | 42 |
| L | L | 25 | 75 |
| BL | B | 13 | 75 |
|  | L | 25 | 69 |
| BBLL | B | 42 | 58 |
|  | L | 63 | 63 |
